# Critical Issues Faced by Industries Associated with Food Science and Technology: A Delphi Analysis

**DOI:** 10.3390/foods13244149

**Published:** 2024-12-21

**Authors:** Kevan W. Lamm, Andrews Idun, Peng Lu

**Affiliations:** Department of Agricultural Leadership, Education & Communication, University of Georgia, Athens, GA 30606, USA; andrews.idun@uga.edu (A.I.); penglu@uga.edu (P.L.)

**Keywords:** food science, critical issue, Delphi, expert panel

## Abstract

As the foundation of human health, the food system is arguably a cornerstone of society. However, despite the criticality of a safe and productive food value chain there are numerous critical issues faced by industries associated with food science and technology. Using a three round Delphi process, this study identified the most critical issues faced by these industries. Based on input from expert panelists representing industry, policy makers, and academics, a total of 120 critical issues were identified in the first round. Through a consensus-building process in two subsequent rounds, 38 issues were retained. The retained issues were then analyzed using the constant comparative method to identify themes. A total of eight themes emerged from the analysis, including the following (alphabetically): (1) education, training, and workforce development; (2) emerging technologies in food sciences; (3) food safety and public health; (4) fresh produce and raw food operations; (5) microbiome and pathogens; (6) product innovation and development; (7) quality assurance and systems management; and (8) sustainability and climate resilience. These results provide a robust foundation to help guide and inform strategic priorities and actions within the food industry.

## 1. Introduction

Food is a fundamental source of nutrition, energy, and sustenance, serving as the foundation for human life and overall well-being [1]. Equally importantly, the food industry spans the agriculture, farming, food production, processing, and retail sectors, serving as an essential component of the global economy [2,3]. The food industry not only drives economic growth and generates employment but also plays a critical role in ensuring food security, food safety, and food product development [4]. From farm to table, the food industry supports the global population and is essential for societal well-being [5]. In addition, the food industry drives food innovations through the development of new food products to provide the global population with safe and nutritious foods [6]. However, these multifaceted contributions come with evolving challenges as food science and technology advance.

In recent years, the food industry has faced significant challenges related to food science and technology [7]. These challenges include the complexities of food product development, ensuring food quality and safety, managing efficient food production systems, and navigating the transformation of raw commodities into consumable products [7]. For instance, when developing new food products, food scientists often face the challenge of formulating ingredients that align with consumers’ preferences and health trends, while balancing taste, texture, and nutritional value [7]. At the same time, they must ensure adherence to food safety regulations and maintain cost-effectiveness in production. For example, in the European Union, there is legislation under consideration which may impact the procurement, processing, and sale of food, specifically, “an EU level intervention will aim to establish new foundations for future food policies by introducing sustainability objectives and principles on the basis of an integrated food system approach” paragraph 2 in [8].

In addition to aligning with policy standards, assessing food quality presents further challenges. For example, traditional methods may overlook critical chemical reactions that impact nutrition, taste, and texture [9]. Simultaneously, emerging techniques like high-pressure processing may unintentionally degrade essential nutrients. To improve quality monitoring, food scientists require advanced tools that integrate multiple scientific approaches, ensuring food remains both safe and nutritious [10]. Key food safety concerns faced by the food industry include microbial and chemical contamination, the vital role of government oversight, and the need for improved communication between regulators and the industry [10]. That study also highlighted the challenges posed by globalization in maintaining consistent safety standards across borders and emphasizes the importance of adopting new technologies, such as genomic sequencing, to enhance safety protocols.

Recently, Yadav et al. [11] identified several challenges faced by the current agricultural food supply chain, including the rising demand for food and increased consumer focus on food safety. Furthermore, sustainability concerns, such as reducing food waste and conserving resources, are prompting food industry professionals to adopt new strategies to design more efficient food logistics networks. In addition, globalization has added new layers of complexity to food supply chains, making it essential for industry leaders to address these challenges to ensure food safety and quality on a global level [12]. Additionally, consumer acceptance and affordability are critical factors to consider when developing food science and technology solutions, particularly with novel food products and technologies. Many consumers are hesitant to trust unfamiliar foods and the perceptions of the risks and benefits associated with new food products differ significantly among consumers, which can further influence their acceptance [13,14].

Despite the recognition of these challenges, there is a limited body of literature that comprehensively identifies and prioritizes the significant issues faced by the food industry associated with food science and technology from a research perspective. Although previous studies have identified various challenges, there is a need for a systematic examination of the overarching challenges across food product development, safety, and production.

Our study adopts the theories of opinion leadership [15] and consensus building [16] as its framework. Opinion leaders are often perceived as credible and trustworthy individuals who play central roles within communication networks [17]. They are often regarded as more knowledgeable and well-informed compared to others in their network. The opinion leadership model generally involves a single individual, known as the opinion leader, who first acquires new information and subsequently disseminates it to the members of their network. Research in agricultural contexts [18] has highlighted the distinct insights of opinion leaders within the industry. These findings align with studies of opinion leadership in health-related fields [17]. Consensus building was employed, as “consensus building can offer more knowledge, ideas, and effective joint action for these conditions than the cleverest policymaker or analyst” [16] (p. 421). Using a social process was intended to ensure a variety of perspectives were considered throughout the process, resulting in a meritocracy of ideas.

Our study aims to bridge an existing gap within the literature; specifically, providing a link between academic research and industry practice. By identifying critical issues, our study aims to inform more appropriate strategic, tactical, and operational efforts. This study was driven by the following research objectives:

1. Create a robust list of potentially critical issues faced by industries associated with food science and technology;
2. Achieve a consensus on the specific critical issues faced by industries associated with food science and technology;
3. Develop a heuristic thematic grouping of critical issues faced by industries associated with food science and technology.

## 2. Materials and Methods

### 2.1. Delphi Process

The Delphi process has been used in several studies to gain consensus among experts on a particular topic (see [19]). It involves asking a series of questions that seek to elicit responses from the participants by using feedback to guide the process. According to Zawacki-Richter [20], one of the essential aspects of the Delphi process is the anonymity it offers participants when sharing their opinions, alleviating the problems caused by a few prestigious or influential individuals dominating a group; thus, improving group decision-making processes by taking advantage of seeking opinions without face-to-face interaction [21].

### 2.2. Expert Panel

The success of a Delphi study is significantly influenced by the selection of the expert panel [22]. Their expertise and diverse perspectives are crucial in shaping the study’s outcomes [19]. For this study, individuals from both academic and industry backgrounds in food science and technology were selected for the expert panel. All expert panelists were from the United States. The external experts panel included 40 individuals from various backgrounds, including large food processing and manufacturing companies and federal regulatory organizations. Most had senior or supervisory level experience within the food industry. Panelists’ titles included Project Scientist, Director of Global Food Safety and Regulation, Microbiologist, and Food Safety Scientist. External panel members were nominated for inclusion based on their expertise within the food industry by academic experts. The internal experts included 27 faculty members from a department of Food Science and Technology at a large public institution.

### 2.3. Data Collection

Three rounds of data collection were completed using an electronic Qualtrics survey [23]. Data were collected from July 2022 to August 2022 and were part of a larger planning project. The survey was sent using the Qualtrics online survey platform. In the first round of the process, conducted in July 2022, the panelists were asked to respond to the following question: “In your opinion, what are the most critical issues facing industries associated with food science and technology (such as food product development, food safety, food production, and so forth)?” The panel members were asked to provide a word or short phrase to describe up to five of the top critical issues briefly. In the second round, panelists were presented with a list of potential critical issues based on the data collected in round one. Having completed round one of the processes, the responses of external and internal panelists were combined. The purpose of including external and academic perspectives was to ensure that a robust set of potential issues could be considered in subsequent process rounds [19].

Based on the overarching planning project, only internal panel members were invited to complete rounds two and three of the process. During round two of the study, respondents were asked to review the issues identified in round one and respond to the following question: “Please indicate the level of importance you associate with each of the following issues industries associated with food science and technology are facing: 1 = Not at all important, 2 = Somewhat important, 3 = Important, 4 = Very important, 5 = Extremely important”. The results from round two were collated and presented to participants in round three. In the third round, panel members were asked to select a list of critical issues faced by the food science and technology industries. Panelists were asked to respond to the following prompt: “Please indicate whether the following issues facing industries associated with food science and technology should be kept or removed from the final list:”. Two possible answer choices, Keep or Remove, were presented for each item. Respondents indicated which critical issues they felt should be kept or removed in the final list. Response rates for each round are displayed in Table 1.

### 2.4. Data Analysis

The data were analyzed using SPSS V. 25 statistical software. Duplicate items were removed, and items generated during round one were consolidated. In round two, expert panel members rated each issue generated in round one according to their level of importance. After calculating the mean scores for each issue [24,25], a threshold score of 3.27 was established a posteriori [26]. Items with a mean importance score greater than or equal to 3.27 were retained. A level of consensus was established in round three. Based on recommendations in the literature, a consensus threshold of 76.92% was determined a posteriori [27], resulting in a final list of 38 issues retained from the Delphi process.

Based on the results of the Delphi process, a constant comparative analysis was conducted on the final list of critical issues [28]. Using this method, researchers can generate heuristic thematic groupings of items based on repeated comparisons (e.g., [19]). At the beginning of the process, each item was examined and categorized into as many categories as possible [29]. To generate themes, the researchers compared each item with previous items coded into the same category [29]. As a result of repeated comparisons, higher-order categories emerged, lending greater insight into data themes [29]. Before transferring the data to an electronic format, codes, themes, and categories were manually analyzed [19]. The researchers then conducted peer debriefings and member checks to reduce bias and ensure that the analysis presented was trustworthy [29].

## 3. Results

In the first round of the Delphi, 120 unique responses were received regarding critical issues in the food science and technology industries. During round two, the expert panelists rated the importance of each issue. Table 2 summarizes the results from round two, including the frequency counts and average scores for each issue.

Following round two of the Delphi process, 66 issues were retained. The third round consisted of expert panelists providing their level of agreement on each issue. Table 3 summarizes the results of round three.

After round three of the Delphi process, 38 issues were identified. These issues were thematically analyzed utilizing the constant comparative method. Eight heuristic themes were identified as a result of the constant comparative method analysis. Table 4 summarizes these themes and their associated issues.

## 4. Discussion

The current study identified numerous critical issues faced by industries associated with food science and technology; however, the panel reached 100% consensus on only three issues. This result indicates there are a limited number of issues which were universally perceived as critical. The critical issues where consensus was achieved through the process were subsumed across eight primary themes: food safety and public health, emerging technologies in food sciences, sustainability and climate resilience, microbiome and pathogens, education, training and workforce development, fresh produce and raw food operations, product innovation and development, quality assurance and systems management. The identification of higher-order themes provides a more coherent framework for food science and technology focus and future actions.

Starting with the education, training, and workforce development theme, the emergence of the theme was somewhat anticipated. Previous research has found many front-line workers in the food industry are usually high school graduates without degrees [30]. These workers are mostly employed in the production environment as supervisors, production managers, operations managers, bakers, slaughterers, meat packers, food batch makers, inspectors, testers, sorters, and samplers, as well as other non-degree-requiring positions in transportation sectors such as moving materials, as well as service occupations such as fast food and counter employees [31]. Even though these workers may not be required to possess a degree in food science, Napoleon et al. [30] contend that they have a direct impact on the efficiency of production, the quality of the products produced, and the safety of food being consumed by consumers. Therefore, it is necessary to provide these workers with the knowledge and skills to adapt to changes (e.g., technology) within the industry more broadly. To this end, a recommendation would be to develop a career technical education (CTE) program designed to assist prospective food science and technology workers in developing a variety of skills, such as developing good safety habits, obtaining a thorough understanding of resources and managing them, utilizing technology effectively, conducting research, and solving problems. The ability to think critically and creatively, communicate efficiently with superiors and colleagues, work effectively as part of a team, and resolve conflicts appropriately are context agnostic and provide a foundation to adapt to ever-changing professional environments [18,32].

Regarding the themes of food safety and public health and microbiome and pathogens, both anticipated and unanticipated issues were identified. For example, it is well established in the literature that globally, food safety has become a major focus in public health due to its impact on people of all ages, races, genders, and socioeconomic levels [33,34]. In many African countries, outbreaks of foodborne diseases, such as cholera, shigellosis, konzo, and acute aflatoxicosis continue to occur [35]. Approximately 300,000 infants and young children in China were affected by the 2008 contamination of infant formula with melamine, of which 51,900 were hospitalized and six children died [36]. While, in the United Kingdom there is a great deal of public concern over foodborne exposure to agricultural and environmental chemicals [37]. This has resulted in public authorities demanding that industries associated with food science and technology develop a comprehensive quality management and food inspection system to improve food safety, enhance consumers’ information, and regain consumers trust in food (e.g., [38]). However, consumers’ behavior, such as eating shellfish raw, and demanding minimal processed foods with long shelf-lives, no preservatives, and low salt are likely to increase the danger of pathogen outbreaks [39]. Kiprotich et al. [40] found that the consumption of food contaminated with foodborne pathogens and microbial by-products could result in serious illnesses and economic losses. An associated recommendation is for industries associated with food science and technology to continue to implement best management practices to minimize food safety concerns and coordinate with policy makers to ensure regulations are appropriate throughout the food value chain [37,41], including in international trade and supply chains [33].

Regarding the theme of fresh produce and raw food operations, the issues identified were somewhat anticipated. For example, studies have shown that fresh produce is highly nutritious, containing water-soluble vitamins and other nutrients that contribute to enhancing nutrition and reducing cardiovascular disease risk in humans [42]. However, since fresh produce is not prepared thoroughly before consumption, contamination with pathogens along the value chain may pose a serious threat to consumers [43]. Therefore, to obtain high-quality fresh products, a recommendation is that harvesting, postharvest handling, transportation and storage procedures should be prioritized amongst food producers. In addition to the quality of the primary commodity, processing and handling factors have a noteworthy impact on the quality of raw material used for fresh-cut products [44]. An additional recommendation would be to train growers and primary processors in the best practices for continuous improvement [45]. Additionally, consumers should be educated on the best way to handle fresh food produce such as the right temperature for storage and preparation before consumption.

In the sustainability and climate resilience theme, the identified issues were somewhat consistent with expectations. The results indicate that sustainability and climate resilience are a major concern in the food industry. According to Garnett [46], even though food is essential to human survival, its production and supply chain are a major cause of greenhouse gas emissions, unsustainable water extraction and pollution, deforestation, and biodiversity loss. As food production is significantly influenced by human behavior, their beliefs and attitudes tend to determine the importance placed on the sustainability of the food supply chain [47]. However, even with a growing segment of environmentally conscious consumers actively attempting to change their purchasing and consumption behaviors, there has been limited evidence that these efforts have had a measurable impact globally [48]. Therefore, to achieve sustainable food production and supply, especially in the context of climate change and the need for climate resilience and resource efficiency, a recommendation is to employ a systems-based approach to integrate essential inputs, processes, and outputs. Specifically, beginning with production processes and moving through the entire supply chain structures and operations to the end consumer may help to ensure more alignment in sustainable production and consumption [49]. Existing research has already indicated that climate-resilient agricultural strategies have the potential to enhance sustainable food production and security globally [50].

Lastly, the remaining themes (product innovation and development, emerging technologies in food sciences, and quality assurance and systems management) included several issues previously established in the literature. For example, there has been a growing trend among industries to use emerging technologies to improve food safety and to extend products’ shelf lives [51]. Among the various food processing technologies, high-pressure processing, microwave heating, and pulsed electric fields were the most common food processing technologies [51,52]. With these technological advancements, food manufacturers have been able to provide many more options than were available years ago [53]. Today, consumers can select from a wide range of options. For example, food and beverage products with reduced caloric density are available in the market based on technological advancements in food production [53]. Like any other industry, the food industry considers product development an integral component of effective business management [54]. Even though studies show that product innovation and development is driven by a technological perspective, market perspective, and consumer-led product development [54], developing food products depends greatly on consumer perceptions and acceptance, and thus including the consumer in the development process is essential to minimize the likelihood of failure [54]. A recommendation is for industries associated with food science and technology to make use of consumer research in their bid to introduce new technology or enter new markets. Similarly, the emergence of artificial intelligence (AI) (e.g., [55,56]), big data (e.g., [57]), and machine learning (e.g., [58,59]) has shown significant promise in food science and technology-related industries. An associated recommendation would be for food-related industries to continue to pilot test technology-based approaches in areas such as consumer demand, pathogen detection and prediction, process optimization, and supply chain management. Limited preliminary adoption may help to improve operational integration and organizational learning, allowing for the eventual scaling of technology adoption in the future. The appropriate adoption of technologies and approaches would be anticipated to have positive impacts on quality and systems management within food-related industries as well. This recommendation is based on existing research which has found inverse relationships between technology adoption in the food system and sustainability [60], thus warranting caution.

### 4.1. Implications for Theory

The results of this study provide empirical data to suggest that the use of opinion leadership [15] and consensus building [16] have the potential to create both robust and comprehensive insights into complex and diverse contexts. Overall, the external panel proposed 37 of the 66 items, or 56%, included in round three of the process. This group also proposed six of the thirteen items (46%) with 90–100% consensus. These results indicate that the external and internal academic panel structure may provide novel insights without necessarily biasing results towards a praxis or academic/theoretical perspective. An associated recommendation would be to ensure that Delphi panels include diverse experts with varied experiences and insights.

### 4.2. Implications for Practice

From a practical perspective, the results of the current study provide a harmonized set of critical issues for the industry. Developing a common set of overarching themes may help to improve the coordination of efforts across groups and organizations. These results also help to establish key focus areas as a discipline, rather than anecdotal areas of interest. A recommendation would be to frame subsequent projects within the context of these primary theme areas to aggregate and scaffold efforts. This also has the potential to improve communication and innovation adoption. As Braken and Oughton [61] state, “common understanding derived from shared languages in turn plays a vital role in enhancing the relations of trust that are necessary for effective interdisciplinary working” (p. 371). An additional recommendation would be for the industry to proactively work towards addressing the critical issues identified through this study and to communicate efforts to diverse stakeholders, including policy makers, regulators, consumers, and so forth. These efforts may help to mitigate policy related regulation impacts and demonstrate a commitment to science-directed continuous improvement by the industry.

### 4.3. Limitations

Despite the novel and comprehensive findings from this study, several limitations are also important to note. First, the results of the study are dependent on the insights and expertise of the included panelists. This is a well-documented limitation associated with the Delphi method. However, “studies have consistently shown that for questions requiring expert judgment, the average of individual responses is inferior to the averages produced by group decision processes” [62] (p. 19). Nevertheless, it is possible there are other critical issues which may exist but were beyond the domains of expertise of the expert panel. An associated recommendation would be to interpret the results accordingly. Additionally, the use of academic panelists for rounds two and three should be acknowledged. It is likely that different issues and themes may have emerged if a different (external) expert panel were to complete the process. Second, the primary themes which emerged from the constant comparative analysis are intended to be heuristic and not necessarily definitive. Although 38 issues are less than the 120 generated in the first round of the process, it is still cognitively challenging to retain this volume of issues. The eight themes which emerged are intended to provide a guide and framework for ongoing dialogue. A recommendation would be to use the emergent themes as a foundation for dialogue using the specific critical issues to inform more tactical actions. Lastly, the pace at which technological and societal changes are occurring must be acknowledged. What were contemporary issues when data were collected and analyzed may have evolved. Therefore, an associated recommendation would be to collect longitudinal data regarding issue persistence among food industry experts. Tracking the emergence and disappearance of issues may help to clarify priorities on a more operational level.

## 5. Conclusions

As the foundation of human health, the food system is arguably a cornerstone of society. However, despite the criticality of a safe and productive food value chain there are numerous critical issues faced by industries associated with food science and technology. The present study presents an aggregation of the most critical issues identified by a panel of experts. Although the results of the study indicate there are a multitude of potential issues (120 were generated in the first round of the process), the 38 which were retained following the third and final round of the process represent a more conceptually manageable number. Extending the consolidation further, the constant comparative method analysis yielded eight primary themes which subsumed the 38 unique critical issues. These eight themes represent a comprehensive set of the most critical issues faced by industries associated with food science and technology. Based on these results, academics, industry professionals, policy makers, and other stakeholders have a common set of focus areas. Aligning efforts and actions with the most critical themes and issues has the potential to improve the food system value chain.

## Figures and Tables

**Table 1:** Delphi expert panel response rates.

Delphi Round	External Panel (n, Invited)	External Panel Response Rate	Internal Panel (n, Invited)	Internal Panel Response Rate
Round 1	30	75%	21	78%
Round 2	n/a	n/a	18	67%
Round 3	n/a	n/a	15	56%

**Table 2:** Delphi round two sum of frequency counts and mean results: level of importance for critical issues faced by industries related to food science and technology (1 = Not at all important, 2 = Somewhat important, 3 = Important, 4 = Very important, 5 = Extremely important).

Issue	1	2	3	4	5	Mean
Food safety (including chemical and toxicological) ^a^	0	0	4	6	5	4.07
Food quality and healthy food	0	0	7	2	6	3.93
Sustainable food production	0	0	5	5	4	3.93
Emerging pathogens	0	2	4	3	6	3.87
Microbiologists/pathologists/virologists with a working knowledge of actual fresh produce operations ^a^	0	0	6	5	4	3.87
Fresh produce outbreaks	1	0	4	5	5	3.87
Sustainability ^a^	0	1	4	5	4	3.86
Shifting mindsets towards systemic food safety ^a^	1	1	2	7	4	3.80
Listeria and other food safety	0	1	5	5	4	3.80
Applicable research (of all kinds) for fresh, raw produce operations ^a^	0	2	4	4	5	3.80
Climate change resilience	1	0	6	1	6	3.79
Scaling a product safely ^a^	1	1	3	6	4	3.73
Supply chain disruption	1	2	2	4	5	3.71
Impact on supply chain	1	1	4	3	5	3.71
In-plant food safety: culture, implementation, sanitary design, consistency ^a^	1	1	3	5	4	3.71
Food insecurity ^a^	0	3	2	5	4	3.71
Big data and AI ^a^	0	1	7	3	4	3.67
Microbiome ^a^	0	1	7	3	4	3.67
Food quality	0	1	6	5	3	3.67
Emerging technologies and scale-up ^a^	1	0	6	4	4	3.67
Food for long-term health ^a^	0	2	4	6	3	3.67
Food security	0	3	2	6	3	3.64
Climate change/drought/water scarcity ^a^	1	1	5	2	5	3.64
Supply chain shortages and cost	1	2	2	5	4	3.64
Artificial Intelligence in Food System	0	2	4	5	3	3.64
Sustainability/green processing technology	2	0	4	3	5	3.64
Training and education ^a^	0	2	5	3	4	3.64
Supply continuity ^a^	1	2	2	4	4	3.62
Quality assurance ^a^	0	1	7	4	3	3.60
Regulatory compliance (food safety, food quality, workforce)	1	1	2	9	1	3.57
Personnel who can communicate/work in the technical details of a food safety program while communicating/working with non-technical people ^a^	2	0	4	4	4	3.57
Machine Learning in food system	0	1	7	3	3	3.57
Reducing environmental footprint of food production ^a^	1	2	4	2	5	3.57
Novel technology acceptance	0	3	4	5	3	3.53
Supply chain challenges ^a^	1	2	3	5	3	3.50
Affordable healthy food	0	2	5	5	2	3.50
In-depth/holistic technical knowledge of food science ^a^	0	2	6	3	3	3.50
Need to integrate automation with food science ^a^	1	1	4	8	1	3.47
Health-promoting food ingredients	0	2	6	5	2	3.47
Food waste ^a^	0	3	4	3	3	3.46
Supply chain/logistics ^a^	1	3	2	3	4	3.46
Food availability ^a^	0	4	4	2	4	3.43
Food production (crops/livestock) affected by climate change ^a^	1	1	6	3	3	3.43
Public understanding of food and nutrition	1	2	4	4	3	3.43
Raw materials, availability, extraction, purification ^a^	0	4	3	4	3	3.43
Genomics in microbial detection of pathogens and spoilage ^a^	0	5	3	3	4	3.40
Food processing technology	0	4	5	2	4	3.40
Price increases ^a^	2	0	5	3	3	3.38
Nutrition and health ^a^	1	3	4	2	4	3.36
Rising food costs ^a^	1	2	5	3	3	3.36
Adequate food supply	1	2	5	3	3	3.36
Emerging trends and products	0	3	5	4	2	3.36
Availability of well-trained employees	3	2	1	3	5	3.36
Ingredients shortage ^a^	1	2	4	5	2	3.36
Product development with novel ingredients ^a^	0	2	8	3	2	3.33
Food preservation and shelf-life	0	1	9	4	1	3.33
Quality systems ^a^	0	2	8	3	2	3.33
Shift to resilient operations—Quality management systems adapting in real-time to varying levels of risk ^a^	1	2	5	5	2	3.33
Supply of materials	1	4	1	4	3	3.31
Dependence on fossil fuels	3	1	3	3	4	3.29
Data analytics—Customers and consumers providing active (e.g., surveys) and passive (e.g., telemetry) feedback on product quality more than ever before	2	1	5	3	3	3.29
Price and availability ^a^	1	3	4	3	3	3.29
Use of diverse and beneficial crops ^a^	0	3	7	3	2	3.27
Technology adoption	0	5	3	5	2	3.27
Use of energy efficient technologies ^a^	1	4	3	4	3	3.27
Minimal processing	2	2	4	4	3	3.27
Competency of employees ^a^	3	2	2	3	4	3.21
Changing consumer perception of certain processes ^a^	1	4	3	3	3	3.21
Need for traditionally trained food microbiologist ^a^	3	1	5	2	4	3.20
Product innovation	0	3	8	2	2	3.20
Attracting and retaining labor ^a^	4	1	2	3	4	3.14
Shifting consumer preference/Customization	1	2	6	4	1	3.14
Public perception of processed/manufactured foods ^a^	2	1	7	1	3	3.14
Transparency-Increased connectivity across customers, suppliers and other partners ^a^	1	4	4	2	3	3.14
Global trends	1	2	6	4	1	3.14
Food manufacturing	0	3	9	1	2	3.13
Diversity in terms of personnel in higher management	3	0	6	3	2	3.07
Production expertise ^a^	1	3	4	6	0	3.07
Lack of consumer education ^a^	2	2	5	3	2	3.07
Changing regulations	1	4	5	3	2	3.07
Alternative proteins in new foods ^a^	0	5	6	2	2	3.07
Trained personnel able to begin work on growing, harvesting, handling, packing, and shipping operations ^a^	1	4	3	6	0	3.00
Fragmentation—global regulatory framework ^a^	0	6	2	4	1	3.00
Achieving/proving sustainability goals without greenwashing	5	0	3	2	4	3.00
Healthy formula foods ^a^	0	4	7	4	0	3.00
Development of compostable packaging	1	4	6	2	2	3.00
Intelligent packaging to preserve foods	1	3	6	5	0	3.00
Food production ^a^	0	5	7	2	1	2.93
Manufacturing capabilities ^a^	1	2	10	1	1	2.93
Developing a new product ^a^	1	4	7	1	2	2.93
Technology shifts—can be directly related to food or auxiliary factors which change the way food is delivered, prepared, etc., to the consumer ^a^	1	4	7	1	2	2.93
Lack of talent in the manufacturing/process engineering space ^a^	5	1	1	4	3	2.93
Packaging sourcing ^a^	1	5	3	4	1	2.93
Inflation ^a^	3	2	4	3	2	2.93
Dwindling number of farms ^a^	3	2	5	1	3	2.93
Meeting consumer demands through new product development (e.g., clean label)	0	4	8	1	1	2.93
Labeling, claims, and other legal/regulatory issues ^a^	1	4	4	5	0	2.93
Increasing demand ^a^	1	4	5	3	1	2.93
Converting chemical to natural processes ^a^	0	6	5	1	2	2.93
Consumer mishandling of foods	2	4	2	3	2	2.92
Clean labeling	2	3	6	3	1	2.87
Alternative protein development	1	4	6	4	0	2.87
Research and development of new products	1	6	5	0	3	2.87
Food product development expertise ^a^	0	4	9	2	0	2.87
Corruption of companies and government agencies	4	3	1	3	3	2.86
Increasingly stringent standards ^a^	2	4	4	2	2	2.86
Digitization ^a^	3	3	2	5	1	2.86
Understanding processes in food production ^a^	1	5	5	1	2	2.86
Undeclared allergens ^a^	1	4	5	4	0	2.86
Labor shortages ^a^	4	2	1	4	2	2.85
Cultured meat/alternative meat products	1	6	5	2	1	2.73
Getting a nutrition label ^a^	2	5	3	3	1	2.71
Food insights ^a^	2	4	5	2	1	2.71
Valorization for additive use	6	0	3	5	1	2.67
Launching a new product ^a^	2	3	8	2	0	2.67
New product development/initiation ^a^	0	6	8	1	0	2.64
Understanding the true purpose of food ^a^	5	1	4	2	2	2.64
Hyper-automation—Everything that can be automated will be automated ^a^	4	3	3	2	2	2.64
Unhealthy consumers—need more nutrition ^a^	4	1	5	4	0	2.64
Speed to product development ^a^	3	6	4	2	0	2.33

^a^ External expert panel-sourced item.

**Table 3:** Delphi round three results: level of consensus for critical issues faced by industries related to food science and technology.

**Issue**	**Consensus (%)**
Genomics in microbial detection of pathogens and spoilage ^a^	100
Food safety (including chemical and toxicological)	100
Emerging pathogens	100
In-plant food safety: culture, implementation, sanitary design, consistency	92.86
Microbiome ^a^	92.86
Machine learning in food system	92.86
Fresh produce outbreaks ^a^	92.86
Food processing technology ^a^	92.86
Artificial intelligence in food system	92.86
Applicable research (of all kinds) for fresh, raw produce operations ^a^	92.86
Training and education	92.31
Regulatory compliance (food safety, food quality, workforce) ^a^	92.31
Sustainability	92.31
Microbiologists/pathologists/virologists with a working knowledge of actual fresh produce operations	85.71
Listeria and other food safety ^a^	85.71
In-depth/ holistic technical knowledge of food science ^a^	85.71
Food waste ^a^	85.71
Food preservation and shelf-life ^a^	85.71
Emerging technologies and scale-up	85.71
Data analytics—Customers and consumers providing active (e.g., surveys) and passive (e.g., telemetry) feedback on product quality more than ever before ^a^	85.71
Availability of well-trained employees ^a^	85.71
Personnel who can communicate/work in the technical details of a food safety program while communicating/working with non-technical people	85.71
Big data and AI ^a^	85.71
Sustainable food production	84.62
Sustainability/green processing technology	84.62
Shifting mindsets towards systemic food safety	84.62
Shift to resilient operations—Quality management systems adapting in real-time to varying levels of risk ^a^	84.62
Raw materials, availability, extraction, purification ^a^	84.62
Emerging trends and products ^a^	83.33
Product development with novel ingredients	78.57
Health-promoting food ingredients ^a^	78.57
Food quality and healthy food	78.57
Climate change resilience ^a^	78.57
Technology adoption	76.92
Quality systems ^a^	76.92
Quality assurance	76.92
Scaling a product safely ^a^	76.92
Reducing environmental footprint of food production ^a^	76.92
Minimal processing	71.43
Impact on supply chain ^a^	71.43
Food security ^a^	71.43
Food quality ^a^	71.43
Food insecurity ^a^	64.29
Climate change/drought/water scarcity	64.29
Use of energy efficient technologies ^a^	61.54
Novel technology acceptance ^a^	61.54
Need to integrate automation with food science	61.54
Public understanding of food and nutrition ^a^	57.14
Affordable healthy food ^a^	57.14
Adequate food supply ^a^	57.14
Supply chain challenges	53.85
Ingredients shortage	53.85
Nutrition and health	50.00
Food for long-term health ^a^	50.00
Food availability ^a^	50.00
Use of diverse and beneficial crops	46.16
Food production (crops/livestock) affected by climate change ^a^	42.86
Price and availability ^a^	35.71
Supply chain disruption	30.77
Dependence on fossil fuels	28.57
Supply of materials	25.00
Supply continuity ^a^	23.08
Supply chain/logistics ^a^	23.08
Price increases	21.43
Rising food costs ^a^	15.38
Supply chain shortages and cost	15.38

^a^ External expert panel-sourced item.

**Table 4:** Constant comparative method thematic analysis results (*n* = 38).

**Issue**	**Number of Issues**	**Number of Issues with 90–100% Agreement**
*Food Safety and Public Health*	9	4
Food quality and healthy food		
Food safety (including chemical and toxicological) ^1^		
Genomics in microbial detection of pathogens and spoilage ^a,1^		
Health-promoting food ingredients ^a^		
In-plant food safety: culture, implementation, sanitary design, consistency ^1^		
Listeria and other food safety ^a^		
Regulatory compliance (food safety, food quality, workforce) ^a,1^		
Shift to resilient operations—Quality management systems adapting in real-time to varying levels of risk ^a^		
Shifting mindsets towards systemic food safety		
*Emerging Technologies in Food Sciences*	7	3
Artificial intelligence in food system ^1^		
Big data and AI ^a^		
Data analytics—Customers and consumers providing active (e.g., surveys) and passive (e.g., telemetry) feedback on product quality more than ever before ^a^		
Emerging technologies and scale-up		
Food processing technology ^a,1^		
Machine learning in food system ^1^		
Technology adoption		
*Sustainability and Climate Resilience*	6	1
Climate change resilience ^a^		
Food waste ^a^		
Reducing environmental footprint of food production ^a^		
Sustainability ^1^		
Sustainability/green processing technology		
Sustainable food production		
*Microbiome and Pathogens*	2	2
Emerging pathogens ^1^		
Microbiome ^a,1^		
*Education, Training, and Workforce Development*	5	1
Availability of well-trained employees ^a^		
In-depth/holistic technical knowledge of food science ^a^		
Microbiologists/pathologists/virologists with a working knowledge of actual fresh produce operations		
Personnel who can communicate/work in the technical details of a food safety program while communicating/working with non-technical people		
Training and education ^1^		
*Fresh Produce and Raw Food Operations*	3	2
Applicable research (of all kinds) for fresh, raw produce operations ^a,1^		
Fresh produce outbreaks ^a,1^		
Raw materials, availability, extraction, purification ^a^		
*Product Innovation and Development*	4	
Emerging trends and products ^a^		
Food preservation and shelf-life ^a^		
Product development with novel ingredients		
Scaling a product safely ^a^		
*Quality Assurance and Systems Management*	2	
Quality assurance		
Quality systems ^a^		

^a^ External expert panel-sourced item, ^1^ Item with 90–100% consensus.

## Data Availability

The original contributions presented in the study are included in the article, further inquiries can be directed to the corresponding author.
